# Marsupial brood care in Cretaceous tanaidaceans

**DOI:** 10.1038/s41598-017-04050-8

**Published:** 2017-06-29

**Authors:** Alba Sánchez-García, Xavier Delclòs, Michael S. Engel, Graham J. Bird, Vincent Perrichot, Enrique Peñalver

**Affiliations:** 1 Departament de Dinàmica de la Terra i de l’Oceà and Institut de Recerca de la Biodiversitat (IRBio), Facultat de Ciències de la Terra, Universitat de Barcelona, Martí i Franquès s/n, 08028 Barcelona, Spain; 2 Division of Invertebrate Zoology, American Museum of Natural History, Central Park West at 79th Street, New York, New York, 10024-5192 USA; 3 Division of Entomology, Natural History Museum, and Department of Ecology & Evolutionary Biology, University of Kansas, 1501 Crestline Drive, Lawrence, Kansas 66045-4415 USA; 4 8 Shotover Grove, 5036 Waikanae, Kāpiti New Zealand; 5 CNRS UMR 6118 Géosciences, Université Rennes 1, Campus de Beaulieu, 263 Avenue du Général Leclerc, 35042 Rennes Cedex, France; 6 Museo Geominero, Instituto Geológico y Minero de España, Ríos Rosas 23, 28003 Madrid, Spain

## Abstract

Parental care in animal evolution has long fascinated biologists, but tracing this complex of behavioural repertoires is challenging, as these transitory states often leave no corporeal traces as fossils. Among modern invertebrates, the tanaidaceans (Malacostraca: Peracarida), a lineage of marsupial crustaceans, show an interesting variety of brooding strategies. Here we report on fossil tanaidaceans from the Cretaceous of Spain and France that provide conclusive evidence for marsupial care of brood-offspring. Two exceptionally preserved female specimens of *Alavatanais carabe* and *A*. *margulisae* from Late Albian Peñacerrada I amber (Spain) possess four pairs of rudimentary oostegites, indicating formation of a marsupium. From Recent data, given the taxonomic distribution of a marsupium of four pairs of oostegites, we hypothesize that this may be plesiomorphic for the Tanaidomorpha. We also report on a peculiar tanaidacean specimen referable to the fossil family Alavatanaidae, *Daenerytanais maieuticus* gen. et sp. nov., from Early Cenomanian La Buzinie amber (France), preserved with its marsupial pouch and content. Our discoveries provide early evidence of the peracarid reproductive strategy, as seen in modern Tanaidacea, and argue that this form of parental care may have played a role in the diversification of the lineage during this period.

## Introduction

The fossil record provides a rich and valuable repository of behavioural and evolutionary developments despite the influence of biases in preservation or density of taxonomic representation. Many behaviours are fleeting, uncommonly leaving behind trace fossils, and are therefore captured by exceptional ‘frozen moments’ or are inferred indirectly from functional morphology. Nonetheless, the behaviours of extinct species are critical to understanding the totality of their lives; for placing them within broader palaeoecological settings; and for revealing intra- and interspecific interactions that were undoubtedly at play. They are also critical for the documentation of evolutionary novelties and trends, and understanding phylogenetic relationships.

The study of parental care is especially important, as its appearance and development are closely linked with other key traits in evolutionary biology^1^. The evolution of parental care also has significant implications for understanding the complex interplay between ecology, life history, and the social environment^2^. Parental care, traditionally dubbed ‘subsociality’, is defined as any trait shown by parents that increases the survival and growth of their offspring, and persists when favouring the survival of the species^1^. The degree of parental care differs widely and is exhibited by a broad range of animal taxa; it varies with respect to duration, as well as the form, level, and the extent to which it is provided by the mother, father, or both parents^3^. Parental care is thought to have evolved independently numerous times among invertebrates^3–5^, including various lineages of crustaceans^6^. A notable example of parental care among the Crustacea is the marsupial Peracarida, a group that includes the order Tanaidacea. As with most of the related amphipods, cumaceans, isopods, and (doubtfully) mysids, female tanaidaceans have a specialized structure in the form of a ventral brood pouch (marsupium) that retains and protects their eggs and embryos until they emerge as mancae, or juveniles (which is also a defining condition of the peracarids)^7^.

The Tanaidacea are a diverse and abundant group of usually small and cryptic crustaceans that, except for some rare freshwater and brackish species, today constitutes an almost entirely marine order that is found at all latitudes and in almost all marine benthic habitats from the littoral to hadal zones^8^. Despite a wealth of information on the taxonomy and phylogeny of these widespread crustaceans, our empirical understanding of their life history and parental care trade-offs is currently limited to a few descriptive reviews^9, 10^, with a few taxon-specific studies^11–16^. Fossil evidence revealing the origin and evolutionary history of their reproductive strategy is lacking, although modern-looking tanaidaceans have recently been discovered in the Cretaceous^17–19^.

The preservation of fossil tanaidaceans is rare, mostly because they have non-recalcitrant tissues and cuticles, and there are extensive gaps present in the fossil record for the order. Fossilised specimens have been dated from as far back as the Lower Carboniferous, with the oldest species being discovered in Scotland^20, 21^. Several rock-impressions have been found, and recent studies identified many tanaidaceans as bioinclusions in Lower–Upper Cretaceous fossil resins from Spain and France^17–19^. From the Cenozoic only four, as-of-yet undescribed specimens have been recorded in Lower Miocene Mexican amber^22^.

Only very rarely does preservation allow sufficient support for inferences about behaviour, or demonstrate such ethologies outright. Examples in the fossil record that suggest parental care are scarce, although popularized by records among Amniota^23–25^. Certainly, the occurrence of demonstrably eusocial (~colonial) lineages as fossils serve indirectly as occurrences of parental investment given the nature of such animal societies^26, 27^, as do the records of fossil nests or nesting materials used to construct brooding chambers^28^, which themselves represent parents actively working to provide a stable and protective microhabitat for the development of offspring. However, other forms of parental investment among what may be termed ‘presocial’ species^29^ are more difficult to document as fossils. Some exceptional examples of parental investment have been recorded among insects^30–34^ and arachnids^35, 36^, but rarely in crustaceans. The only unequivocal cases of brooding among crustaceans are the mid-Silurian *Nymphatelina gravida* Siveter *et al*. from the Herefordshire Konservat-Lagerstätte in England^37^, the Upper Ordovician *Luprisca incuba* Siveter *et al*., from the Katian Stage Lorraine Group^38^, and the Early Miocene *Aquitanoscia chiapasensis* Broly *et al*., in press and *A*. *maternus* Broly *et al*., in press, from the Aquitanian Chiapas amber^39^, the former two myodocope ostracods and the latter two isopods. Other evidence of reproductive strategies in fossil crustaceans are otherwise indirect (refs 37 and 40 and references therein), or are restricted to putative *in situ* eggs or embryos of a bradoriid species^41, 42^, a waptiid^43^, a tealliocaridid^44^, a syncarid^45^, two branchiopod species^46, 47^, and a few other ostracods^48^.

Recent discoveries in Spanish and French ambers provide unique evidence of parental care in tanaidaceans from the Cretaceous period, confirming the order’s long history of this behavioural/life-history adaptation. Indirect evidence from paired structures preserved in minute detail on the coxae of two fossil species from the Late Albian of the Peñacerrada I outcrop (Spain) — *Alavatanais carabe* Vonk & Schram, and *A*. *margulisae* Sánchez-García, Peñalver & Delclòs — implies the development of a specialized marsupium for carrying offspring. Direct evidence is provided by a new paratanaoidean species, assigned to the extinct family Alavatanaidae Vonk & Schram, from the Early Cenomanian La Buzinie outcrop (France), which is preserved with a cluster of eggs within a marsupium. These discoveries extend our knowledge of the palaeobiology of the group, and document, for the first time, the presence of tanaidaceans in the La Buzinie amber. These species confirm that this complex reproductive strategy, still present in modern Tanaidacea, existed in their ancient relatives almost 105 million years ago, thus indicating considerable constancy in brooding development and behaviour over this expanse of geological time and evolutionary space.

## Results

**Figure 1:**
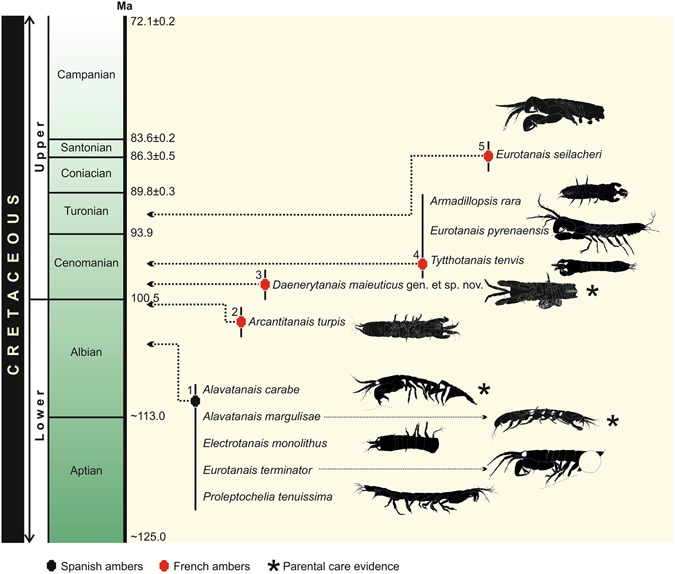
Timeline of fossil tanaidomorphans described from amber. 1 = Peñacerrada (Upper Albian), note that only one specimen of *Alavatanais carabe* was found in the El Soplao outcrop (Upper Albian), 2 = Archingeay/Les Nouillers (Upper Albian), 3 = Champniers/La Buzinie (Early Cenomanian), 4 = Fourtou (Middle Cenomanian), and 5 = La Garnache (Turonian). Numerical ages are from^70^.

Recent discoveries in the Cretaceous ambers of Spain and France have revealed an unexpected diversity and abundance of Tanaidacea, showing that this period was significant in the diversification and evolution of the order^18, 19^. Earlier accounts of Spanish and French amber tanaidaceans reported a total of seven genera and ten species which has now grown by one new genus and species from La Buzinie (Charente, France) (Fig. 1). All the Cretaceous species known to date are members of the Tanaidomorpha (one of two suborders in Tanaidacea; the former Neotanaidomorpha and Anthracocaridomorpha are no longer recognized). Extant tanaidomorphans are characterized by having a ventral marsupium of variable conformation, but overall formed by one or four pairs of oostegites^49^.

**Figure 2:**
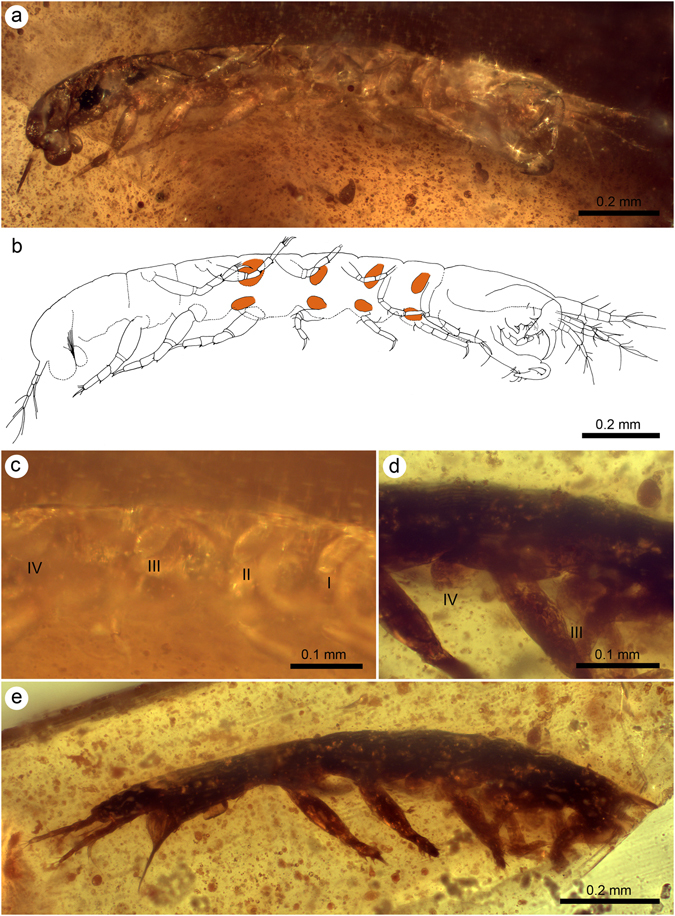
Female alavatanaids from the Lower Cretaceous amber of Peñacerrada I, Spain. (**a**) Lateral overview of *Alavatanais margulisae* (holotype MCNA 9583a) showing the oostegites; (**b**) Camera lucida drawing of the specimen in A highlighting the oostegites in orange (modified from Sánchez-García *et al*.^18^); (**c**) Detail of right oostegites I–IV of the same specimen; (**d**) Detail of the third and fourth right oostegites of *A*. *carabe* (MCNA 13890); (**e**) Lateral overview of the same specimen of *A*. *carabe* showing the oostegites.

Low sexual dimorphism described for *Alavatanais carabe* from the two morphs preserved was based on differences in size, number of antennular articles (four- or five-articled in females versus seven-articled in males), and robustness of the cheliped^18^. However, the most remarkable character shared by females of *A*. *carabe* and *A*. *margulisae* is the presence of pairs of bud-like developing oostegites at the coxal plates of pereopods I–IV, as found in related extant ‘preparatory’ females, which eventually expand to become more laminar and complete the marsupium during the copulatory stage. The oostegites appear as inwardly directed, medium-sized, pear-shaped structures of an average length and width of 0.07 and 0.03 mm in *A*. *margulisae*, and 0.08 and 0.04 mm in *A*. *carabe* (Fig. 2).

Other amber-preserved species known from isolated specimens were potentially assigned as males or females based on secondary sexual characters such as the number and/or shape of the antennular articles, and from modifications of the male cheliped^18^. Greatly lengthened chelipeds with the shape of the chelae altered — the dactylus and propodus having a convex curvature resulting in a central gap when the chela is closed — were reported for males of the genus *Eurotanais* Sánchez-García, Peñalver & Delclòs and *Alavatanais* Vonk & Schram. The only reported functions for this enlargement of the male cheliped in the recent fauna are to tear open female mucous tubes^11^ or to fight with other males^50^.

The new genus from the Early Cenomanian of La Buzinie is unambiguously referred to Paratanaoidea, as evidenced by (1) its general habitus; (2) antennule with five or fewer articles in females (and often with more than five articles in males, although the male is unknown in the present fossil species); (3) antenna with seven or fewer articles; (4) presence of an ischium on all pereopods; and (5) pleon never with the two last pleonites fused/reduced (and always with pleopods in males, although these may be reduced: unknown for the present fossil species as males remain to be discovered)^49^. The large compound eyes, unreduced pereonites I–III, short pleon with five free pleonites not fused with the pleotelson, antennules with four to five articles (in females), pereopod coxa present on all pereopods, pereopod I with medium-long dactylus, dactylus and unguis of pereopods IV–VI claw-like but not fused, and the pleopods well-developed with long setae bundled together all support inclusion to the family Alavatanaidae despite the lack of preservation for the posterior region of the body.

## Systematic Palaeontology

Class Malacostraca Latreille, 1802

Superorder Peracarida Calman, 1904

Order Tanaidacea Dana, 1849

Suborder Tanaidomorpha Sieg, 1980

Superfamily Paratanaoidea Lang, 1949

Family Alavatanaidae Vonk & Schram (*sensu* Sánchez-García *et al*.^18^)

Genus *Daenerytanais* gen. nov.

### Type species

*Daenerytanais maieuticus* sp. nov.

### Etymology

The generic name is a matronym for Daenerys Targaryen, a principal character in the popular fantasy novel series *A Song of Ice and Fire* by George R.R. Martin, alluding to her principal role as the mother of dragons; and combined with *Tanais* Latreille (presumably taken from the ancient Greek city in the Maeotian marshes of the same name), an early generic name used widely as a suffix in the Tanaidomorpha and as the typical genus for the order. The gender of the name is, however, masculine following the precedent of the genus *Tanais*.

### Diagnosis

#### Female

Body relatively slender, about five times longer than wide. Cephalothorax subtriangular when viewed dorsally (much longer than wide), with a lateral constriction beyond its midlength. Antennule with five articles. Antenna short and slender, with subequal articles, never square. Cheliped somewhat robust; propodus with fixed finger deflexed almost perpendicular to palm; dactylus directed medially, extending beyond fixed finger. Pereon rather short (about 0.5 times body length). Pereopod I with long dactylus plus unguis (not longer than propodus); pereopods II–III with dactylus plus unguis much shorter than in pereopod I; pereopods IV–VI armed with weak spines, with dactylus plus unguis as long as in pereopods II–III but stouter. Pleon rather short (less than 0.3 times body length). Male: *Latet*.

**Figure 3:**
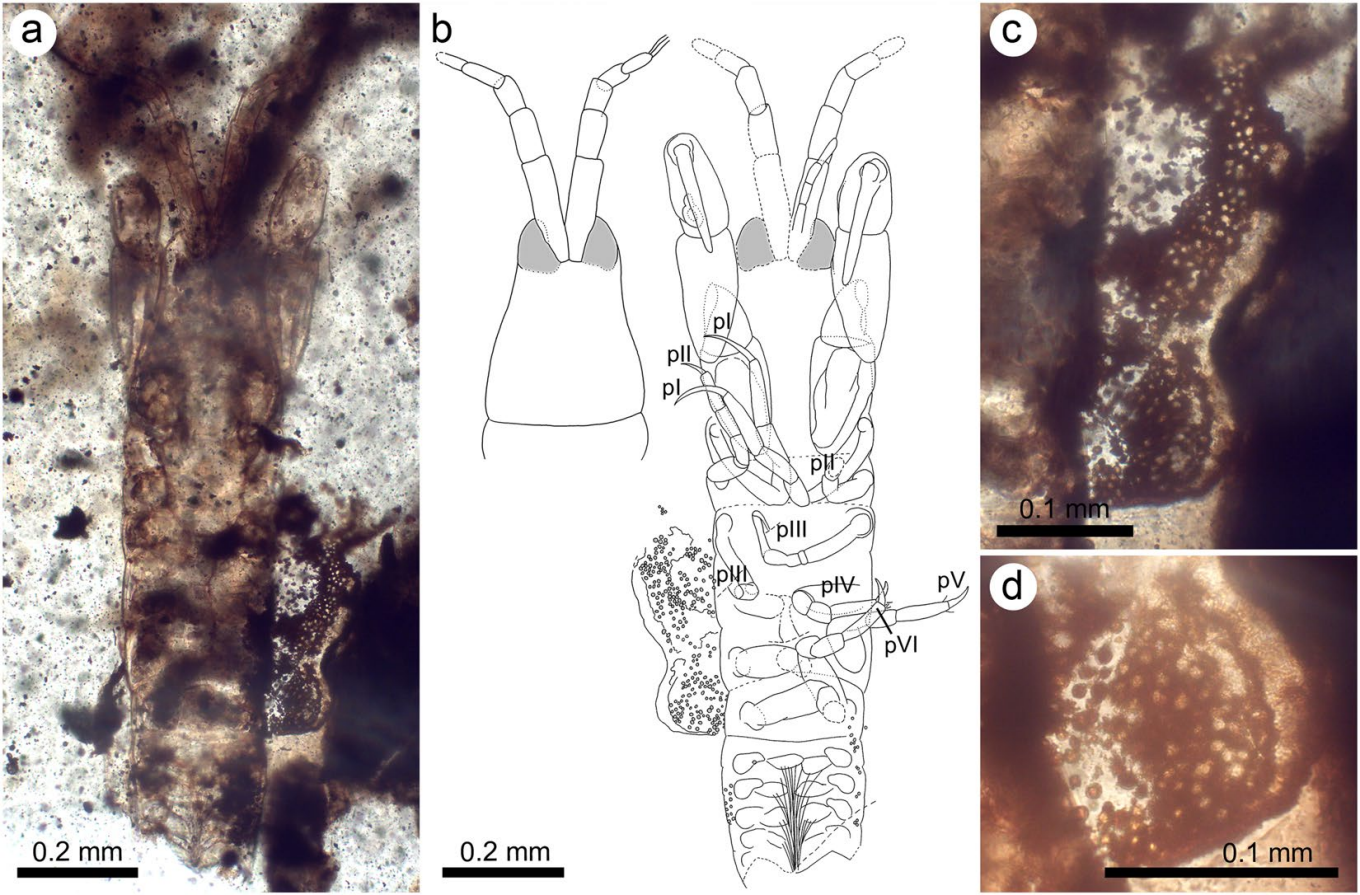
Holotype (IGR.BUZ-1.13), female, of *Daenerytanais maieuticus* gen. et sp. nov., from the mid-Cretaceous French amber of La Buzinie. (**a**) Dorsal habitus. (**b**) Camera lucida drawings of the cephalothorax in dorsal view (left), and the ventral habitus (right). (**c**) Detail of the marsupium. (**d**) Eggs. Abbreviations: pI–pVI = pereopods I–VI.

*Daenerytanais maieuticus* sp. nov. (Fig. 3).

### Etymology

The specific epithet is taken from the Greek *maieutikos*, ‘skilled in midwifery’, and refers to the possession of a developed marsupium for the care of offspring.

### Diagnosis

As for the genus (*vide supra*).

### Material

Holotype and only known specimen IGR.BUZ-1.13, ♀. (Coll. Couillard, housed in the Geological Department and Museum of the University Rennes 1, France). The specimen, nearly complete except for the uropods, is embedded in a small piece of light-yellow amber with multiple bubbles and slightly clouded by organic debris. It can be observed in dorsal and ventral views but not in profile, and thus some chelipedal characters are not currently visible. Some pereopods are badly preserved or cut beyond the basis; the pattern of setation is difficult to distinguish without optimal lighting and magnification.

### General description of the amber piece

Specimen IGR.BUZ-1.13 was originally part of a larger piece (IGR.BUZ-1) containing many fossils and divided into 20 individual components for study. The original set of syninclusions comprised 12 dolichopodid flies (Microphorinae: *Microphorites deploegi* Nel *et al*.)^51^, two schizopterid bugs (*Buzinia couillardi* Perrichot *et al*.)^52^, one scale insect, four scelionid wasps, one roach, one centipede (*Buziniphilus antiquus* Edgecombe *et al*.)^53^, four entomobryomorphan springtails, five prostigmatid mites (Acari: Parasitengona), one isolated nematode, and several amoebae and diatoms^54, 55^.

### Occurrence

La Buzinie outcrop, in Champniers near Angoulême, department of Charente, Nouvelle-Aquitaine Region, southwestern France; Early Cenomanian (amber level A2a)^56^.

### Description

#### Female

Body medium-sized, estimated total length approximately 1.20 mm, about 4.59 times longer than wide; subcylindrical, slightly flattened dorsoventrally. All observed setae simple.

Cephalothorax (head and first two thoracomeres) subtriangular in dorsal aspect, narrowing anteriorly, with a lateral constriction beyond its midlength, 1.32 times longer than maximum width; about 0.29 times total body length, nearly as long as combined lengths of pereonites I–IV; posterior margin rounded, laterally swollen. Rostrum absent. Compound eyes well-developed, large, diameter 0.25 times cephalothorax length, slightly bulging, positioned antero-laterally on cephalothorax and abutting peduncles of antennules. Antennule five-articled, fairly slender, tapering in width distally, slightly longer than cephalothorax (1.18 times length); article I about 0.43 times length of antennule, reaching combined lengths of articles II and III, 3.54 times as long as thick, slightly expanded laterally at cephalothorax insertion, without discernible setae; article II about 0.45 times length of article I, nearly twice as long as thick (1.87 times), without discernible setae; article III about 0.62 times length of article II, 1.38 times as long as thick, without discernible setae; article IV slightly shorter than article III (0.95 times), 1.60 times as long as thick, without discernible setae; terminal article (article V) as long as preceding article but thinner, about as long as thick (1.06 times), with bluntly rounded apex, apex bearing three simple setae, setae nearly as long as article V. Antenna largely obscured, with at least four articles, approximately half length of antennule and much thinner, approximately half thickness of article I of antennule, apically extending at most to basal half of article II of antennule; visible articles subequal in size, about 2.31 times longer than thick, without discernible setae. Mouthparts not visible in holotype as preserved. Cheliped somewhat robust, without discernible setae (most likely an artefact of preservation); sclerite not visible; basis widening distally, 1.85 times longer than wide, 0.87 times length of carpus; merus subtriangular; carpus widening distally, over twice as long as wide (2.22 times), 1.33 times length of propodus; propodus about twice as long as wide (2.10 times), with fixed finger deflexed almost perpendicular to palm; fixed finger and dactylus apparently unequal in length; incisive margin of fixed finger not discernible as preserved (details of fixed finger not observable owing to fossilization position); dactylus directed medially, extending beyond fixed finger, gradually curving along length and tapering in width, with acutely rounded apex, unguis not discernible.

Pereon (the six thoracomeres after the cephalothorax) rather short, about 0.45 of total body length; all pereonites wider than long, with apical margins weakly convex dorsomedially; pereonites I–III subequal in size, about 3.49 times wider than long; pereonites IV and V largest segments, subequal in size, about 2.23 times wider than long, each about 1.53 times individual lengths of pereonites I–III; pereonite VI shorter than preceding pereonite, 2.98 times wider than long, about as long as individual lengths of pereonites I–III (1.01 times). Pereopods I–III without discernible setae; coxae present on all pairs; basis fairly slender, cylindrical, 7.05 times longer than thick, longer than combined lengths of merus and carpus; ischium short (only visible on right pereopod II and left pereopod III); merus and carpus subequal in size, not widening distally; propodus longer than carpus, 4.03 times longer than thick, tapering distally; dactylus plus unguis curved and long on pereopod I (0.88 times length of propodus), becoming shorter on pereopods II and III, (0.45 times length of dactylus plus unguis I). Pereopods IV–VI similar in length to pereopods I–III but stouter; coxae present on all pairs; basis fairly robust, more inflated than in pereopods I–III, not measurable in length as preserved; ischium short (only visible on left pereopod IV); merus and carpus subequal in size; propodus longer than carpus, 3.89 times longer than thick, tapering distally, with up to two distal spines (weak as preserved); dactylus plus unguis claw-like but not fused (only visible on right pereopods IV–VI), as long as dactylus plus unguis of pereopods II and III, but stouter. Marsupium present and filled with eggs (as preserved); eggs around 6–9 microns in diameter.

Pleon rather short, about 0.28 times total body length, slightly tapering in width distally, with five free, subequal pleonites, each bearing a pair of pleopods; pleonites as wide as pereonites but distinctly shorter (each about 0.41 times individual lengths of pereonites IV and V), about 4.96 times wider than long. Pleotelson not preserved. Pleopods alike; basal article rounded, without discernible setae; endopod and exopod subovate, with long terminal setae bundled together under pleon (difficult to enumerate as preserved). Uropods not preserved. Male: *Latet*.

### Remarks

This species displays some similar characteristics to the genus *Alavatanais* (*i*.*e*., the subtriangular cephalothorax and female antennule with four to five articles). Overall, the species is like a female of *A*. *carabe* as a result of the lateral constriction in the cephalothorax beyond its midlength, the rather short pereon (less than 0.5 times the body length), the antennule with five articles, pereopods I–III with a very long dactylus (about 0.9 times the length of the propodus), and pereopods IV–VI possessing a dactylus much shorter and stouter than in pereopods I–III (but armed with weaker spines than in *A*. *carabe*). The chelipedal morphology is somewhat obscured owing to the preservation of the holotype (only visible in dorsal and ventral orientation), but apparently differs from the genus *Alavatanais* in its sturdiness and higher development of the dactylus with respect to the fixed finger. This combination of traits is sufficient to warrant placement of the present species within its own genus, but the two genera may be closely related and this should be tested eventually in a cladistic framework along with other living and fossil tanaidaceans.

Unfortunately, the uropodal configuration remains unknown, so it is impossible to determine if the individual possessed a highly segmented uropod, a putatively plesiomorphic characteristic of alavatanaids. Other distinguishing characters of alavatanaids that should be regarded as plesiomorphies retained from ancestral forms are the presence of coxae on pereopods IV–VI, the unfused claws of the posterior pereopods, the unfused and non-expanded maxilliped endites (unknown for *D*. *maieuticus*), and the free posterior margin of the cheliped basis reaching the first pereonite^18, 19^. It remains to be determined by future cladistic work whether or not the family Alavatanaidae is paraphyletic, perhaps forming a grade to one or more modern families, as the primary features for the family at present are plesiomorphies. It will be important to locate further material for *D*. *maieuticus*, ideally including males, from which the species may be more fully characterized and its generic diagnosis refined relative to *Alavatanais* and other groups.

## Discussion

Parental care evolved to enhance the fitness and survival of the offspring, not uncommonly at the expense of the parents, although any (parental) cost can be seen to be mitigated by the increased contribution to the species’ population^1^. Parental care in crustaceans is reported from all major environments, and different lineages scattered across the diversity of the clade^6^. It ranges from a minimum of care to a wide variety of elaborate behaviours of which the most common are brooding and attendance of both eggs and offspring. In some cases of brooding, females carry their offspring on their body (often in special structures) until they reach advanced larval or fully developed juvenile stages. These forms of parental investment create many opportunities and problems that are different from those of crustaceans that release their eggs directly into the water to become pelagic larval stages^6^.

Among lineages with direct parental involvement, sexually mature females of the peracarid Tanaidacea have a highly specialized structure in the form of a ventral brood pouch (marsupium) in which they care for the offspring after hatching, feeding and protecting the young as they grow and until they emerge as mancae, or juveniles. Apart from the Tanaidacea, specialized marsupia occur as an inferred synapomorphy in the other groups of the Peracarida^7^, with the exception of the thermosbaenaceans that use the carapace to brood the developing embryos—this trait apparently evolved convergently to improve offspring growth and survival.

In the Tanaidacea, the female marsupium is generally composed of paired structures (oostegites) arising from the coxae of the pereopods that overlap to form a brood pouch on the pereon venter. These paired structures are large, thin-walled, concave plates, and their location and number vary within the superfamily Paratanaoidea (suborder Tanaidomorpha). As shown by the two female specimens of *A*. *carabe* and *A*. *margulisae* with paired oostegites preserved, these paired structures are borne on pereopod coxae I–IV, a conformation characteristic of most paratanaoids. Yet apomorphic modifications of this ground-plan do occur within the superfamily. In the paratanaoid family Pseudotanaidae there is a reduction of the anterior oostegites such that the marsupium comprises a single pair of marsupial plates originating from pereopods IV^57^. The single pair of marsupial plates has been considered tied to the reduction in length of pereonites I–III relative to pereonites IV–VI^49, 58^. This could be interpreted as a ‘cost-saving’ measure in development, although empirical demonstration of this is lacking and other ontogenetic factors that lead to segment reduction cannot be excluded. In the remaining families of Paratanaoidea, the four pairs of oostegites, as seen in the amber fossils, probably represent the plesiomorphic condition for the superfamily given the spread of their taxonomic occurrence, but this hypothesis should be tested in future phylogenetic treatments. It is notable that in the superfamily Tanaoidea (family Tanaididae), the marsupial state is achieved by one pair of ovisacs borne on the coxae of pereopod IV; these ovisacs are structurally different from oostegites^13, 15^. Frequently, only one ovisac is developed either from one of the pereopods IV^12^, with no discernible handedness, or is coalesced from both left and right coxae (Bird, pers. obs. on *Sinelobus* Sieg, 1980 and *Zeuxo* Templeton, 1840 spp. at least).

**Figure 4:**
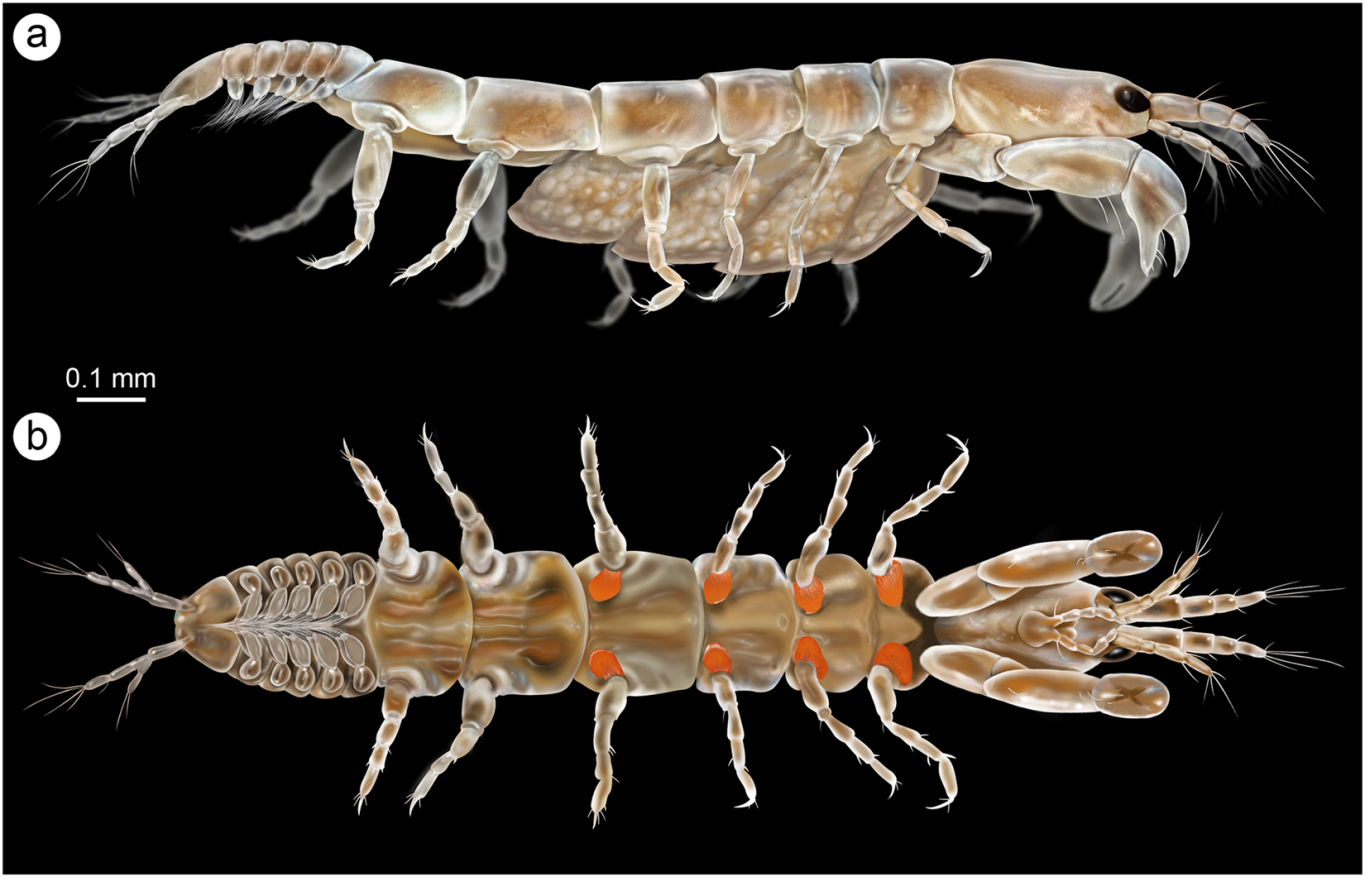
Reconstruction of an adult female of *Alavatanais margulisae* based on the holotype from Peñacerrada I amber (Álava, Spain), and to show its habitus in two different developmental stages. (**a**) Hypothetical ovigerous stage in lateral habitus. (**b**) Preparatory stage in ventral habitus. Illustrations by J.A. Peñas with scientific supervision.

As with most crustaceans, female tanaidaceans undergo a variable number of developmental stages before reaching sexual maturity^16^. Maturation directly influences oostegite morphology and, whereas the early instars always show these structures as ovoid flattened outgrowths, later instars experience a progressive enlargement and thinning of the oostegites. The oostegite development observed in both amber specimens of *A*. *carabe* and *A*. *margulisae* corresponds to those of preparatory females, and the oostegites would further increase in size during subsequent moults to form an entire marsupium (Fig. 4). This is an advanced immature stage intercalated between the earliest developmental stages and the copulatory females with fully-developed marsupia, as is the case observed for *Daenerytanais maieuticus*.

Although there is no record of tanaidaceans bearing oostegites from French ambers, our discovery of a female of *D*. *maieuticus* preserved with its marsupial pouch and contents constitutes a unique example of direct evidence of a specialized egg-brooding strategy among ancient Tanaidacea. The fossil appears to be a copulatory stage with a fully developed marsupium filled with eggs. The number of ova seems excessive, with over 200 eggs preserved. However, the extant typhlotanaid *Peraeospinosus magnificus* (Kudinova-Pasternak, 1970) has been recorded with over 80 eggs in its maternal burrow (tube-like lining), although some of those eggs were possibly unfertilized and intended as food for the mancae, as has been reported previously in *Tanais dulongii* (Audouin, 1826). Indeed, unfertilized eggs are consistent with the small size observed in the fossil. The marsupium, quite voluminous, is clustered near or in contact with the pereon in its normal position but somewhat laterally and posteriorly offset (Fig. 3a,b). Despite the exceptional nature of this find, the lack of more specimens ideally representing various developmental stages leaves open the question of how the marsupium was ultimately formed in *D*. *maieuticus*. However, the fact that pereonites I–III are not reduced, and the apparent affinities of the species with *Alavatanais*, suggest that the marsupium of *D*. *maieuticus* is likely to have developed from four pairs of progressively enlarging and thinning oostegites.

Even though brood care has been posed as an extended reproductive strategy across the Crustacea and has evolved independently multiple times, fossil evidence of such behavioural repertoires is poorly known within the clade, and has only been unequivocally documented in two female myodocope ostracods, *Nymphatelina gravida* and *Luprisca incuba*, retaining eggs and possible juveniles in a brood space^37, 38^; and two comparatively modern Miocene isopods^39^. The egg-brooding reproductive mode for Ostracoda (particularly myodocopes) is coupled with morphological specializations such as a posteriorly inflated carapace, and is completely different from those specializations which enable the ventrally-suspended marsupium of peracarids.

The present fossils are the first fossil evidence for the unique brooding strategies and specialized marsupia of the Tanaidacea; they attest that such relevant adaptations associated with considerable maternal investment were already well established by the Cretaceous. The origin of the peracarid marsupium must date back at least to the Lower Carboniferous (Visean) (*ca*. 340 Ma) from where the earliest anthracocarid tanaidacean fossils were recorded, and presumably much earlier to the common ancestor of the peracarid clade. Our data also tend to support the hypothesis that a marsupium formed by four pairs of oostegites is plesiomorphic for paratanaoidean tanaidomorphans owing to the distribution of this trait across known taxa, although this must be tested by future cladistic analyses. Habitat structure, environmental conditions, food sources or predation pressure have been proposed as motive factors important for the origin and evolution of parental care^59^. The degree of parental care and lack of a free-living larval stage typify the generalized niche to which a group belongs. In this respect, the marsupium represents a safe environment for the offspring and has certainly contributed to the success of Tanaidacea (as well as amphipods and isopods) in diverse habitats, including marine, freshwater, and even moist-terrestrial environments, as has been proposed for some tanaidaceans found in Cretaceous ambers^18, 19^. French and Spanish amber deposits largely represent a sampling of taxa from above the forest floor but also from the soil and litter and even nearby aquatic habitats^60–62^. In fact, there are various other arthropods together preserved with the tanaidaceans that are indicators of a litter-dwelling to semi-aquatic fauna^18, 19^. It is evident that brood care, greatly increasing the offsprings’ survivorship, could therefore have been a significant driver for the diversification and success of tanaidaceans during the Cretaceous.

## Material and Methods

The Spanish specimens (repository numbers MCNA 13890 and MCNA 9583a; housed at Museo de Ciencias Naturales de Álava, Vitoria-Gasteiz, Spain) from the Peñacerrada I site, in Burgos Province, were isolated within small pieces of transparent amber, then embedded in regular prisms of epoxy resin (EPO-TEK 301) under vacuum, and finally ground and polished with a water-fed flat lap for optimal viewing and curation^63^. The geological setting of the Peñacerrada I locality has been previously outlined^64^, and the fossiliferous resins of these sediments have been recently dated as Upper Albian^65^. To date, a broad spectrum of biological inclusions has been recovered, and the vast majority of these represent terrestrial arthropod lineages^66^.

The new French specimen (repository number IGR.BUZ-1.13; housed at the Geological Department and Museum of the University Rennes 1, France) originates from the La Buzinie site, in Charente. The amber piece containing the specimen studied here comes from the lowermost stratum, the lithological level A2a, dated as Early Cenomanian^52, 56, 67^. Several arthropods and protists were fossilized together with the tanaidacean in the same piece, and the amber matrix was rather turbid, which made examination of the inclusion difficult. Therefore, the amber was reworked using a scalpel as a microsaw to approach the tanaidacean as closely as possible; the fossil tanaidacean, separated from its syninclusions, was embedded in Canada Balsam between cover slips^68^.

Examination of the fossils used both Motic BA310 and Olympus BX41 compound microscopes, and measurements were taken with the Image J software package and recorded in millimetres. Photomicrography was performed with a Moticam 2500 digital camera attached to the Motic BA310 compound microscope with Motic Images Plus 2.0 software, at the Universitat de Barcelona (Barcelona, Spain). The software package Helicon Focus was used to combine different focal layers. Line drawings were made under incident and transmitted light with the aid of a camera-lucida attached to the Olympus BX41 compound microscope at the Instituto Geológico y Minero de España (Madrid, Spain). Drawings were then inked and scanned into Adobe Photoshop CS3. A detailed reconstruction of *A*. *margulisae* was undertaken to depict its likely aspect in life. Morphological terminology for the description is generally based on^69^, except in the use of Roman numerals to indicate segment number for serially homologous sclerites, thereby avoiding confusion with metrics and counts used in the descriptions. New taxonomic actions are registered with ZooBank under LSID urn:lsid:zoobank.org:pub:7CF345BD-8D8B-4C7E-8960-A24D6D29507B.
